# 3-[(Furan-2-yl­methyl­idene)amino]-1-(4-methyl­phen­yl)thio­urea

**DOI:** 10.1107/S1600536811000110

**Published:** 2011-01-08

**Authors:** Yan-Ling Zhang, Fu-Juan Zhang, Zhi-Hong Xu, Feng-Ling Yang

**Affiliations:** a College of Chemistry and Chemical Engineering, Xuchang University, Xuchang, Henan Province 461000, People’s Republic of China

## Abstract

There are two independent mol­ecules in the asymmetric unit of the title compound, C_13_H_13_N_3_OS, which was obtained from a condensation reaction of *N*-(*p*-tol­yl)hydrazinecarbothio­amide and furfural. The dihedral angles between the mean planes of the tolyl ring and the (furan-2-yl­methyl­ene)hydrazine unit are 39.83 (8) and 48.95 (7)° in the two mol­ecules. The mol­ecules both exhibit an *E* configuration. In the crystal, inter­molecular N—H⋯N and N—H⋯S hydrogen bonds connect the two independent mol­ecules.

## Related literature

For biological applications of thio­semicarbazones, see: Okabe *et al.* (1993 ▶); Hu *et al.* (2006 ▶). For related structures, see: Zhang *et al.* (2005 ▶); Shan *et al.* (2006 ▶).
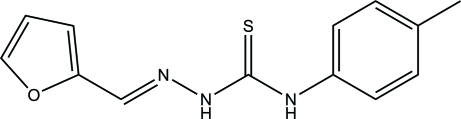


## Experimental

### 

#### Crystal data

C_13_H_13_N_3_OS
*M* *_r_* = 259.32
Monoclinic, 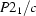

*a* = 12.9464 (3) Å
*b* = 13.8613 (3) Å
*c* = 16.6155 (5) Å
β = 118.028 (2)°
*V* = 2632.01 (12) Å^3^
*Z* = 8
Cu *K*α radiation
μ = 2.12 mm^−1^
*T* = 293 K
0.20 × 0.20 × 0.20 mm

#### Data collection

Oxford Diffraction Xcalibur Eos Gemini diffractometer
Absorption correction: multi-scan (*CrysAlis PRO*; Oxford Diffraction, 2010 ▶) *T*_min_ = 0.677, *T*_max_ = 0.677
19253 measured reflections
4697 independent reflections
3878 reflections with *I* > 2σ(*I*)
*R*_int_ = 0.042

#### Refinement

*R*[*F* ^2^ > 2σ(*F* ^2^)] = 0.043
*wR*(*F* ^2^) = 0.147
*S* = 1.03
4697 reflections
328 parameters
H-atom parameters constrained
Δρ_max_ = 0.19 e Å^−3^
Δρ_min_ = −0.24 e Å^−3^

### 

Data collection: *CrysAlis PRO* (Oxford Diffraction, 2010 ▶); cell refinement: *CrysAlis PRO*; data reduction: *CrysAlis PRO*; program(s) used to solve structure: *SHELXS97* (Sheldrick, 2008 ▶); program(s) used to refine structure: *SHELXL97* (Sheldrick, 2008 ▶); molecular graphics: *SHELXTL* (Sheldrick, 2008 ▶); software used to prepare material for publication: *SHELXL97*.

## Supplementary Material

Crystal structure: contains datablocks I, global. DOI: 10.1107/S1600536811000110/kp2296sup1.cif

Structure factors: contains datablocks I. DOI: 10.1107/S1600536811000110/kp2296Isup2.hkl

Additional supplementary materials:  crystallographic information; 3D view; checkCIF report

## Figures and Tables

**Table 1:** Hydrogen-bond geometry (Å, °)

*D*—H⋯*A*	*D*—H	H⋯*A*	*D*⋯*A*	*D*—H⋯*A*
N2—H2⋯S1*A*^i^	0.86	2.59	3.4397 (19)	171
N2*A*—H2*A*⋯S1^ii^	0.86	2.66	3.3696 (17)	141
N3*A*—H3*A*⋯N1*A*	0.86	2.20	2.628 (2)	111

Symmetry codes: (i) 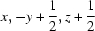; (ii) 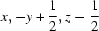.

**Fig. 1.**
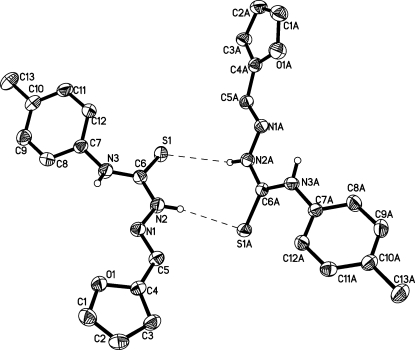
The molecular structure of the title compound, with displacement ellipsoids drawn at 30% probability level. The two molecules of the asymmetric unit are connected by hydrogen bonds N-H···S (dashed lines).

## supplementary crystallographic information

### Comment

Thiosemicarbazones have attracted our attention because of their biological applications (Okabe *et al.*, 1993; Hu *et al.*, 2006). A few single-crystal structres (Zhang *et al.*, 2005; Shan *et al.*, 2006) were reported. For understanding their anticancer activity, it is necessary to have detailed information on their molecular geometries. Both molecules of the asymmetric unit (I) (Fig. 1) reveal an E-configuration. these molecules are related by a pseudo-inversion symmetry. The dihedral angles between the mean planes of the tolyl ring and the (furan-2-ylmethylene)hydrazine unit are 39.83 (8) and 48.95 (7)°. A dominant motif in  a crystal packing are hydrogen bonded dimers via intermolecular N(2)—H(2)···S(1 A)and  N(2 A)—H(2 A)···S(1), interactions (Table 1 and Fig. 1). Intramolecular hydrogen bond N3A-H3A···N1A is observed (Table 1) whereas the other molecule of asymmetric unit does not meet the angle criterium (N-H···N angle is 108 °) for intramolecular hydrogen bond. The value of this angle might be affected by lower accuracy of hydrogen atom position or slight difference between  molecular conformations of these two molecules.

### Experimental

*N*-(*p*-Tolyl)hydrazinecarbothioamide (1.8 g,10 mmol) and furfural (0.96 g, 10 mmol) was dissolved in 95% ethanol (15 mL) and the solution was refluxed for 2.5 h. Fine colourless crystals appeared on cooling. They were filtered and washed by 95% ethanol to give 2.06 g of the title compound in 79.5% yield. Single crystals of (I) were obtained by recrystallisation from acetone.

### Refinement

H atoms were placed in calculated positions with C—H = 0.93–0.96 and N—H = 0.86 Å, and refined using a riding model, *U*_iso_(H) = 1.5*U*_eq_(C) for methyl H atoms and 1.2*U*_eq_(C,*N*).

### Crystal data

**Table:**

C_13_H_13_N_3_OS	*F*(000) = 1088
*M**_r_* = 259.32	*D*_x_ = 1.309 Mg m^−^^3^
Monoclinic, *P*2_1_/*c*	Cu *K*α radiation, λ = 1.54184 Å
Hall symbol: -P 2ybc	Cell parameters from 7546 reflections
*a* = 12.9464 (3) Å	θ = 3.0–72.2°
*b* = 13.8613 (3) Å	µ = 2.12 mm^−^^1^
*c* = 16.6155 (5) Å	*T* = 293 K
β = 118.028 (2)°	Prismatic, colorless
*V* = 2632.01 (12) Å^3^	0.20 × 0.20 × 0.20 mm
*Z* = 8	

### Data collection

**Table:**

Oxford Diffraction Xcalibur Eos Gemini diffractometer	4697 independent reflections
Radiation source: fine-focus sealed tube	3878 reflections with *I* > 2σ(*I*)
graphite	*R*_int_ = 0.042
ω scans	θ_max_ = 67.1°, θ_min_ = 3.9°
Absorption correction: multi-scan (*CrysAlis PRO*; Oxford Diffraction, 2010)	*h* = −15→15
*T*_min_ = 0.677, *T*_max_ = 0.677	*k* = −16→16
19253 measured reflections	*l* = −19→13

### Refinement

**Table:**

Refinement on *F*^2^	Secondary atom site location: difference Fourier map
Least-squares matrix: full	Hydrogen site location: inferred from neighbouring sites
*R*[*F*^2^ > 2σ(*F*^2^)] = 0.043	H-atom parameters constrained
*wR*(*F*^2^) = 0.147	*w* = 1/[σ^2^(*F*_o_^2^) + (0.1*P*)^2^] where *P* = (*F*_o_^2^ + 2*F*_c_^2^)/3
*S* = 1.03	(Δ/σ)_max_ = 0.001
4697 reflections	Δρ_max_ = 0.19 e Å^−^^3^
328 parameters	Δρ_min_ = −0.24 e Å^−^^3^
0 restraints	Extinction correction: *SHELXL97* (Sheldrick, 1997), Fc^*^=kFc[1+0.001xFc^2^λ^3^/sin(2θ)]^-1/4^
Primary atom site location: structure-invariant direct methods	Extinction coefficient: 0.0044 (4)

### Special details

**Table:**

Experimental. CrysAlisPro, Oxford Diffraction Ltd., Version 1.171.34.40 (release 27-08-2010 CrysAlis171 .NET) (compiled Aug 27 2010,11:50:40) Empirical absorption correction using spherical harmonics, implemented in SCALE3 ABSPACK scaling algorithm.
Geometry. All e.s.d.'s (except the e.s.d. in the dihedral angle between two l.s. planes) are estimated using the full covariance matrix. The cell e.s.d.'s are taken into account individually in the estimation of e.s.d.'s in distances, angles and torsion angles; correlations between e.s.d.'s in cell parameters are only used when they are defined by crystal symmetry. An approximate (isotropic) treatment of cell e.s.d.'s is used for estimating e.s.d.'s involving l.s. planes.
Refinement. Refinement of *F*^2^ against ALL reflections. The weighted *R*-factor *wR* and goodness of fit *S* are based on *F*^2^, conventional *R*-factors *R* are based on *F*, with *F* set to zero for negative *F*^2^. The threshold expression of *F*^2^ > σ(*F*^2^) is used only for calculating *R*-factors(gt) *etc*. and is not relevant to the choice of reflections for refinement. *R*-factors based on *F*^2^ are statistically about twice as large as those based on *F*, and *R*- factors based on ALL data will be even larger.

### Fractional atomic coordinates and isotropic or equivalent isotropic displacement parameters (Å^2^)

**Table:**

	*x*	*y*	*z*	*U*_iso_*/*U*_eq_	
S1	0.12468 (5)	0.04591 (4)	0.57517 (3)	0.05268 (19)	
S1A	0.30556 (5)	0.53789 (4)	0.36034 (4)	0.0548 (2)	
O1	0.63171 (13)	0.26407 (12)	0.80209 (9)	0.0551 (4)	
O1A	−0.24494 (16)	0.36883 (14)	0.18097 (11)	0.0676 (5)	
N1	0.42256 (15)	0.16907 (13)	0.73490 (11)	0.0482 (4)	
N1A	−0.02089 (16)	0.45411 (12)	0.24339 (12)	0.0466 (4)	
N2	0.32088 (16)	0.11669 (14)	0.70435 (12)	0.0526 (4)	
H2	0.3089	0.0811	0.7415	0.063*	
N2A	0.08617 (16)	0.48994 (14)	0.25989 (11)	0.0488 (4)	
H2A	0.0953	0.5122	0.2153	0.059*	
N3	0.26010 (16)	0.18718 (14)	0.56597 (12)	0.0534 (4)	
H3	0.3172	0.2263	0.5953	0.064*	
N3A	0.15430 (16)	0.45539 (14)	0.40998 (12)	0.0516 (4)	
H3A	0.0848	0.4331	0.3913	0.062*	
C1	0.7393 (2)	0.3023 (2)	0.85542 (18)	0.0656 (6)	
H1	0.7772	0.3453	0.8351	0.079*	
C1A	−0.3614 (3)	0.3434 (2)	0.12705 (19)	0.0734 (7)	
H1A	−0.4045	0.3037	0.1453	0.088*	
C2	0.7830 (2)	0.2704 (2)	0.93994 (16)	0.0684 (7)	
H2B	0.8552	0.2864	0.9884	0.082*	
C2A	−0.4018 (2)	0.3844 (2)	0.04573 (17)	0.0697 (7)	
H2AA	−0.4772	0.3789	−0.0025	0.084*	
C3	0.6988 (2)	0.20724 (19)	0.94272 (15)	0.0600 (6)	
H3B	0.7047	0.1737	0.9932	0.072*	
C3A	−0.3098 (2)	0.43732 (19)	0.04610 (15)	0.0568 (5)	
H3AA	−0.3126	0.4738	−0.0019	0.068*	
C4	0.60805 (18)	0.20553 (15)	0.85729 (13)	0.0456 (4)	
C4A	−0.2166 (2)	0.42593 (15)	0.12823 (14)	0.0488 (5)	
C5	0.49801 (19)	0.15645 (15)	0.81887 (13)	0.0478 (5)	
H5	0.4809	0.1150	0.8550	0.057*	
C5A	−0.10118 (19)	0.46325 (15)	0.16071 (14)	0.0475 (5)	
H5A	−0.0833	0.4958	0.1199	0.057*	
C6	0.23996 (18)	0.12135 (15)	0.61564 (13)	0.0457 (4)	
C6A	0.17739 (18)	0.49072 (14)	0.34471 (13)	0.0434 (4)	
C7	0.19719 (18)	0.19935 (14)	0.46945 (14)	0.0468 (4)	
C7A	0.22866 (19)	0.45001 (15)	0.50532 (14)	0.0463 (5)	
C8	0.2592 (2)	0.20326 (18)	0.42067 (16)	0.0570 (5)	
H8	0.3405	0.1996	0.4512	0.068*	
C8A	0.1803 (2)	0.4686 (2)	0.56153 (17)	0.0674 (7)	
H8A	0.1016	0.4851	0.5362	0.081*	
C9	0.2013 (2)	0.21261 (18)	0.32708 (17)	0.0622 (6)	
H9	0.2443	0.2155	0.2954	0.075*	
C9A	0.2462 (3)	0.4631 (2)	0.65487 (17)	0.0696 (7)	
H9A	0.2114	0.4764	0.6915	0.084*	
C10	0.0806 (2)	0.21771 (16)	0.27934 (15)	0.0572 (6)	
C10A	0.3629 (2)	0.43844 (17)	0.69498 (15)	0.0535 (5)	
C11	0.0199 (2)	0.21777 (18)	0.32891 (16)	0.0602 (6)	
H11A	−0.0612	0.2233	0.2983	0.072*	
C11A	0.41067 (19)	0.41712 (16)	0.63797 (14)	0.0508 (5)	
H11	0.4888	0.3987	0.6634	0.061*	
C12	0.07673 (19)	0.20985 (17)	0.42347 (15)	0.0550 (5)	
H12A	0.0341	0.2116	0.4555	0.066*	
C12A	0.34502 (19)	0.42261 (16)	0.54403 (14)	0.0485 (5)	
H12	0.3790	0.4079	0.5071	0.058*	
C13	0.0164 (3)	0.2217 (2)	0.17613 (17)	0.0784 (8)	
H13D	0.0540	0.2675	0.1551	0.118*	
H13E	−0.0632	0.2410	0.1560	0.118*	
H13F	0.0177	0.1591	0.1519	0.118*	
C13A	0.4365 (3)	0.4347 (2)	0.79706 (17)	0.0710 (7)	
H13A	0.3925	0.4598	0.8255	0.106*	
H13B	0.5058	0.4728	0.8149	0.106*	
H13C	0.4581	0.3691	0.8159	0.106*	

### Atomic displacement parameters (Å^2^)

**Table:**

	*U*^11^	*U*^22^	*U*^33^	*U*^12^	*U*^13^	*U*^23^
S1	0.0500 (3)	0.0630 (3)	0.0400 (3)	−0.0190 (2)	0.0169 (2)	−0.0050 (2)
S1A	0.0495 (3)	0.0691 (4)	0.0465 (3)	−0.0145 (2)	0.0231 (2)	−0.0043 (2)
O1	0.0478 (8)	0.0680 (10)	0.0420 (7)	−0.0072 (7)	0.0149 (6)	0.0030 (6)
O1A	0.0630 (11)	0.0806 (12)	0.0538 (9)	−0.0012 (9)	0.0230 (8)	0.0119 (8)
N1	0.0433 (9)	0.0527 (9)	0.0410 (8)	−0.0064 (7)	0.0135 (7)	−0.0019 (7)
N1A	0.0440 (9)	0.0521 (9)	0.0412 (9)	0.0009 (7)	0.0178 (8)	0.0003 (7)
N2	0.0482 (10)	0.0609 (10)	0.0411 (9)	−0.0140 (8)	0.0148 (8)	0.0005 (7)
N2A	0.0448 (9)	0.0615 (10)	0.0390 (8)	−0.0020 (8)	0.0188 (7)	0.0046 (7)
N3	0.0454 (9)	0.0605 (11)	0.0422 (9)	−0.0160 (8)	0.0107 (7)	0.0014 (7)
N3A	0.0401 (9)	0.0709 (12)	0.0401 (9)	−0.0100 (8)	0.0156 (8)	0.0018 (7)
C1	0.0520 (13)	0.0771 (16)	0.0630 (14)	−0.0173 (12)	0.0232 (11)	−0.0052 (12)
C1A	0.0677 (16)	0.0854 (18)	0.0701 (16)	−0.0179 (14)	0.0349 (14)	0.0011 (14)
C2	0.0456 (12)	0.0907 (19)	0.0490 (12)	−0.0128 (12)	0.0056 (10)	−0.0063 (12)
C2A	0.0501 (13)	0.0953 (19)	0.0528 (13)	−0.0161 (13)	0.0151 (11)	−0.0055 (12)
C3	0.0540 (13)	0.0713 (14)	0.0393 (10)	−0.0026 (11)	0.0091 (9)	0.0042 (10)
C3A	0.0462 (12)	0.0749 (14)	0.0402 (10)	−0.0061 (11)	0.0127 (9)	0.0084 (10)
C4	0.0442 (11)	0.0475 (10)	0.0390 (10)	0.0015 (8)	0.0145 (8)	−0.0006 (8)
C4A	0.0487 (11)	0.0518 (11)	0.0423 (10)	0.0035 (9)	0.0183 (9)	−0.0001 (8)
C5	0.0480 (11)	0.0466 (10)	0.0431 (10)	−0.0004 (9)	0.0168 (9)	0.0010 (8)
C5A	0.0450 (11)	0.0540 (11)	0.0390 (10)	0.0036 (8)	0.0161 (9)	0.0031 (8)
C6	0.0438 (10)	0.0498 (10)	0.0411 (10)	−0.0050 (8)	0.0181 (8)	−0.0058 (8)
C6A	0.0444 (10)	0.0465 (10)	0.0398 (9)	0.0004 (8)	0.0201 (8)	−0.0028 (8)
C7	0.0434 (10)	0.0448 (10)	0.0435 (10)	−0.0038 (8)	0.0132 (9)	0.0027 (8)
C7A	0.0431 (10)	0.0542 (11)	0.0392 (10)	−0.0071 (8)	0.0172 (9)	−0.0004 (8)
C8	0.0431 (11)	0.0670 (14)	0.0569 (12)	0.0036 (10)	0.0203 (10)	0.0062 (10)
C8A	0.0452 (12)	0.109 (2)	0.0491 (13)	0.0111 (12)	0.0232 (11)	0.0067 (12)
C9	0.0703 (15)	0.0666 (14)	0.0552 (13)	0.0088 (12)	0.0341 (12)	0.0059 (11)
C9A	0.0627 (15)	0.105 (2)	0.0467 (13)	0.0066 (14)	0.0306 (12)	0.0004 (12)
C10	0.0662 (14)	0.0475 (11)	0.0450 (11)	0.0019 (10)	0.0155 (10)	0.0034 (9)
C10A	0.0554 (13)	0.0565 (12)	0.0429 (11)	−0.0058 (10)	0.0183 (10)	−0.0015 (9)
C11	0.0450 (12)	0.0625 (13)	0.0554 (13)	−0.0003 (10)	0.0090 (10)	0.0104 (10)
C11A	0.0441 (11)	0.0530 (11)	0.0490 (11)	0.0009 (9)	0.0166 (9)	0.0041 (9)
C12	0.0434 (11)	0.0656 (13)	0.0532 (12)	−0.0014 (10)	0.0204 (10)	0.0076 (10)
C12A	0.0465 (11)	0.0553 (11)	0.0440 (10)	0.0015 (9)	0.0217 (9)	0.0022 (8)
C13	0.095 (2)	0.0716 (16)	0.0469 (13)	0.0048 (15)	0.0153 (13)	0.0059 (11)
C13A	0.0754 (17)	0.0839 (17)	0.0437 (12)	−0.0012 (14)	0.0197 (12)	−0.0006 (11)

### Geometric parameters (Å, °)

**Table:**

S1—C6	1.682 (2)	C4—C5	1.430 (3)
S1A—C6A	1.686 (2)	C4A—C5A	1.427 (3)
O1—C1	1.359 (3)	C5—H5	0.9300
O1—C4	1.363 (3)	C5A—H5A	0.9300
O1A—C4A	1.353 (3)	C7—C12	1.385 (3)
O1A—C1A	1.389 (3)	C7—C8	1.385 (3)
N1—C5	1.285 (3)	C7A—C8A	1.371 (3)
N1—N2	1.375 (2)	C7A—C12A	1.384 (3)
N1A—C5A	1.281 (3)	C8—C9	1.379 (3)
N1A—N2A	1.373 (2)	C8—H8	0.9300
N2—C6	1.350 (3)	C8A—C9A	1.377 (4)
N2—H2	0.8599	C8A—H8A	0.9300
N2A—C6A	1.348 (3)	C9—C10	1.382 (4)
N2A—H2A	0.8600	C9—H9	0.9300
N3—C6	1.336 (3)	C9A—C10A	1.377 (4)
N3—C7	1.427 (3)	C9A—H9A	0.9300
N3—H3	0.8600	C10—C11	1.380 (4)
N3A—C6A	1.345 (3)	C10—C13	1.515 (3)
N3A—C7A	1.417 (3)	C10A—C11A	1.385 (3)
N3A—H3A	0.8600	C10A—C13A	1.506 (3)
C1—C2	1.320 (4)	C11—C12	1.391 (3)
C1—H1	0.9300	C11—H11A	0.9300
C1A—C2A	1.326 (4)	C11A—C12A	1.385 (3)
C1A—H1A	0.9300	C11A—H11	0.9300
C2—C3	1.415 (4)	C12—H12A	0.9300
C2—H2B	0.9300	C12A—H12	0.9300
C2A—C3A	1.397 (3)	C13—H13D	0.9600
C2A—H2AA	0.9300	C13—H13E	0.9600
C3—C4	1.352 (3)	C13—H13F	0.9600
C3—H3B	0.9300	C13A—H13A	0.9600
C3A—C4A	1.340 (3)	C13A—H13B	0.9600
C3A—H3AA	0.9300	C13A—H13C	0.9600
			
C1—O1—C4	106.22 (18)	N3A—C6A—S1A	126.32 (16)
C4A—O1A—C1A	105.83 (19)	N2A—C6A—S1A	118.65 (15)
C5—N1—N2	115.88 (17)	C12—C7—C8	119.1 (2)
C5A—N1A—N2A	114.36 (17)	C12—C7—N3	122.2 (2)
C6—N2—N1	119.67 (17)	C8—C7—N3	118.73 (19)
C6—N2—H2	120.2	C8A—C7A—C12A	118.7 (2)
N1—N2—H2	120.1	C8A—C7A—N3A	117.6 (2)
C6A—N2A—N1A	121.19 (16)	C12A—C7A—N3A	123.58 (19)
C6A—N2A—H2A	119.4	C9—C8—C7	120.4 (2)
N1A—N2A—H2A	119.5	C9—C8—H8	119.8
C6—N3—C7	126.95 (18)	C7—C8—H8	119.8
C6—N3—H3	116.5	C7A—C8A—C9A	121.1 (2)
C7—N3—H3	116.5	C7A—C8A—H8A	119.4
C6A—N3A—C7A	128.95 (18)	C9A—C8A—H8A	119.4
C6A—N3A—H3A	115.5	C8—C9—C10	121.4 (2)
C7A—N3A—H3A	115.5	C8—C9—H9	119.3
C2—C1—O1	111.0 (2)	C10—C9—H9	119.3
C2—C1—H1	124.5	C10A—C9A—C8A	121.2 (2)
O1—C1—H1	124.5	C10A—C9A—H9A	119.4
C2A—C1A—O1A	109.7 (2)	C8A—C9A—H9A	119.4
C2A—C1A—H1A	125.1	C11—C10—C9	117.7 (2)
O1A—C1A—H1A	125.1	C11—C10—C13	120.8 (2)
C1—C2—C3	106.9 (2)	C9—C10—C13	121.5 (3)
C1—C2—H2B	126.5	C9A—C10A—C11A	117.6 (2)
C3—C2—H2B	126.5	C9A—C10A—C13A	121.6 (2)
C1A—C2A—C3A	107.0 (2)	C11A—C10A—C13A	120.9 (2)
C1A—C2A—H2AA	126.5	C10—C11—C12	121.8 (2)
C3A—C2A—H2AA	126.5	C10—C11—H11A	119.1
C4—C3—C2	106.2 (2)	C12—C11—H11A	119.1
C4—C3—H3B	126.9	C12A—C11A—C10A	121.6 (2)
C2—C3—H3B	126.9	C12A—C11A—H11	119.2
C4A—C3A—C2A	107.5 (2)	C10A—C11A—H11	119.2
C4A—C3A—H3AA	126.3	C7—C12—C11	119.5 (2)
C2A—C3A—H3AA	126.3	C7—C12—H12A	120.3
C3—C4—O1	109.7 (2)	C11—C12—H12A	120.3
C3—C4—C5	132.0 (2)	C7A—C12A—C11A	119.75 (19)
O1—C4—C5	118.33 (17)	C7A—C12A—H12	120.1
C3A—C4A—O1A	109.9 (2)	C11A—C12A—H12	120.1
C3A—C4A—C5A	128.6 (2)	C10—C13—H13D	109.5
O1A—C4A—C5A	121.45 (19)	C10—C13—H13E	109.5
N1—C5—C4	120.63 (19)	H13D—C13—H13E	109.5
N1—C5—H5	119.7	C10—C13—H13F	109.5
C4—C5—H5	119.7	H13D—C13—H13F	109.5
N1A—C5A—C4A	123.2 (2)	H13E—C13—H13F	109.5
N1A—C5A—H5A	118.4	C10A—C13A—H13A	109.5
C4A—C5A—H5A	118.4	C10A—C13A—H13B	109.5
N3—C6—N2	115.50 (18)	H13A—C13A—H13B	109.5
N3—C6—S1	124.88 (16)	C10A—C13A—H13C	109.5
N2—C6—S1	119.62 (16)	H13A—C13A—H13C	109.5
N3A—C6A—N2A	115.00 (18)	H13B—C13A—H13C	109.5
			
C5—N1—N2—C6	−176.10 (19)	C7A—N3A—C6A—S1A	0.8 (3)
C5A—N1A—N2A—C6A	176.01 (19)	N1A—N2A—C6A—N3A	−0.8 (3)
C4—O1—C1—C2	0.2 (3)	N1A—N2A—C6A—S1A	−178.76 (15)
C4A—O1A—C1A—C2A	−0.7 (3)	C6—N3—C7—C12	53.4 (3)
O1—C1—C2—C3	−0.2 (3)	C6—N3—C7—C8	−128.4 (3)
O1A—C1A—C2A—C3A	0.3 (4)	C6A—N3A—C7A—C8A	140.9 (3)
C1—C2—C3—C4	0.1 (3)	C6A—N3A—C7A—C12A	−42.4 (3)
C1A—C2A—C3A—C4A	0.2 (3)	C12—C7—C8—C9	−3.2 (4)
C2—C3—C4—O1	0.0 (3)	N3—C7—C8—C9	178.4 (2)
C2—C3—C4—C5	−178.9 (2)	C12A—C7A—C8A—C9A	2.0 (4)
C1—O1—C4—C3	−0.1 (3)	N3A—C7A—C8A—C9A	178.8 (3)
C1—O1—C4—C5	179.0 (2)	C7—C8—C9—C10	−0.4 (4)
C2A—C3A—C4A—O1A	−0.6 (3)	C7A—C8A—C9A—C10A	−0.3 (5)
C2A—C3A—C4A—C5A	177.9 (2)	C8—C9—C10—C11	3.0 (4)
C1A—O1A—C4A—C3A	0.8 (3)	C8—C9—C10—C13	−176.1 (2)
C1A—O1A—C4A—C5A	−177.9 (2)	C8A—C9A—C10A—C11A	−1.5 (4)
N2—N1—C5—C4	178.51 (19)	C8A—C9A—C10A—C13A	178.5 (3)
C3—C4—C5—N1	177.4 (2)	C9—C10—C11—C12	−2.1 (4)
O1—C4—C5—N1	−1.5 (3)	C13—C10—C11—C12	177.1 (2)
N2A—N1A—C5A—C4A	176.32 (19)	C9A—C10A—C11A—C12A	1.6 (4)
C3A—C4A—C5A—N1A	174.2 (2)	C13A—C10A—C11A—C12A	−178.3 (2)
O1A—C4A—C5A—N1A	−7.4 (3)	C8—C7—C12—C11	4.1 (3)
C7—N3—C6—N2	171.9 (2)	N3—C7—C12—C11	−177.6 (2)
C7—N3—C6—S1	−7.4 (3)	C10—C11—C12—C7	−1.5 (4)
N1—N2—C6—N3	−7.8 (3)	C8A—C7A—C12A—C11A	−1.8 (3)
N1—N2—C6—S1	171.49 (16)	N3A—C7A—C12A—C11A	−178.48 (19)
C7A—N3A—C6A—N2A	−177.0 (2)	C10A—C11A—C12A—C7A	0.0 (3)

### Hydrogen-bond geometry (Å, °)

**Table:**

*D*—H···*A*	*D*—H	H···*A*	*D*···*A*	*D*—H···*A*
N2—H2···S1A^i^	0.86	2.59	3.4397 (19)	171
N2A—H2A···S1^ii^	0.86	2.66	3.3696 (17)	141
N3A—H3A···N1A	0.86	2.20	2.628 (2)	111

Symmetry codes: (i) *x*, −*y*+1/2, *z*+1/2; (ii) *x*, −*y*+1/2, *z*−1/2.
